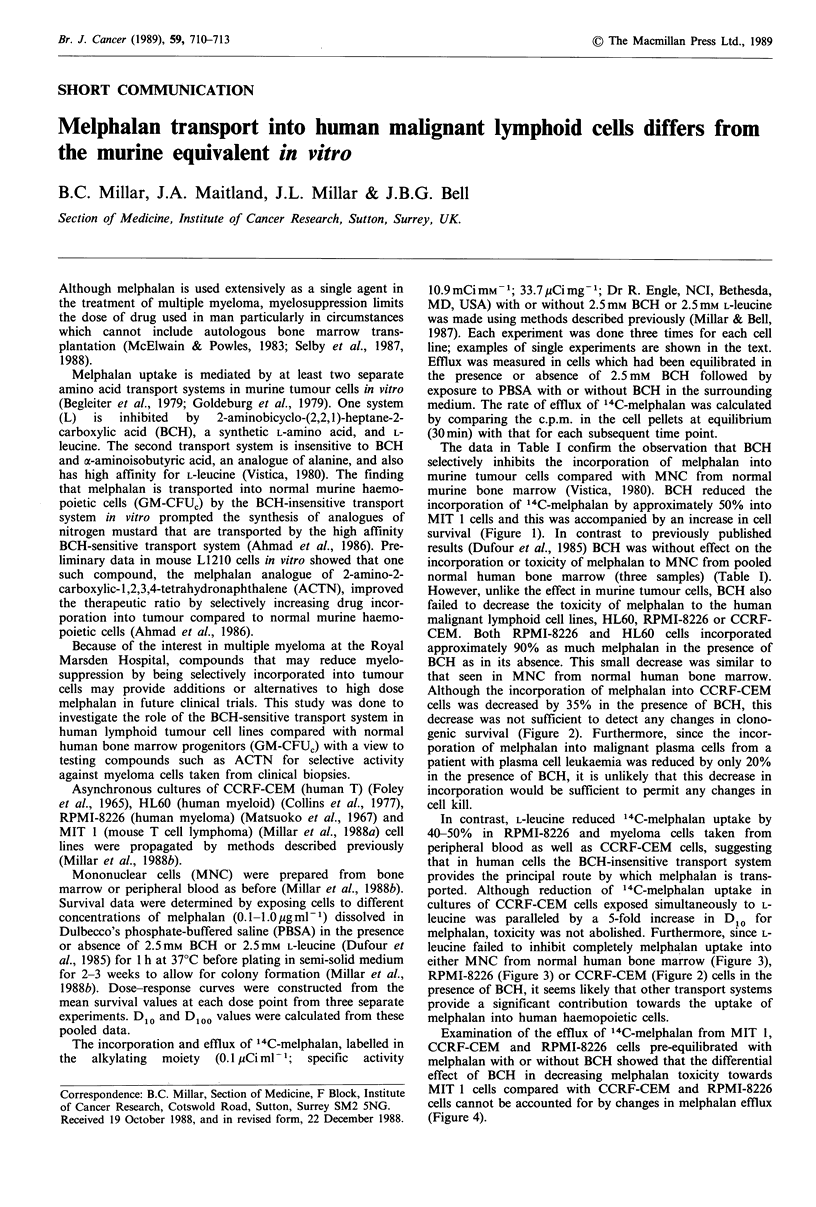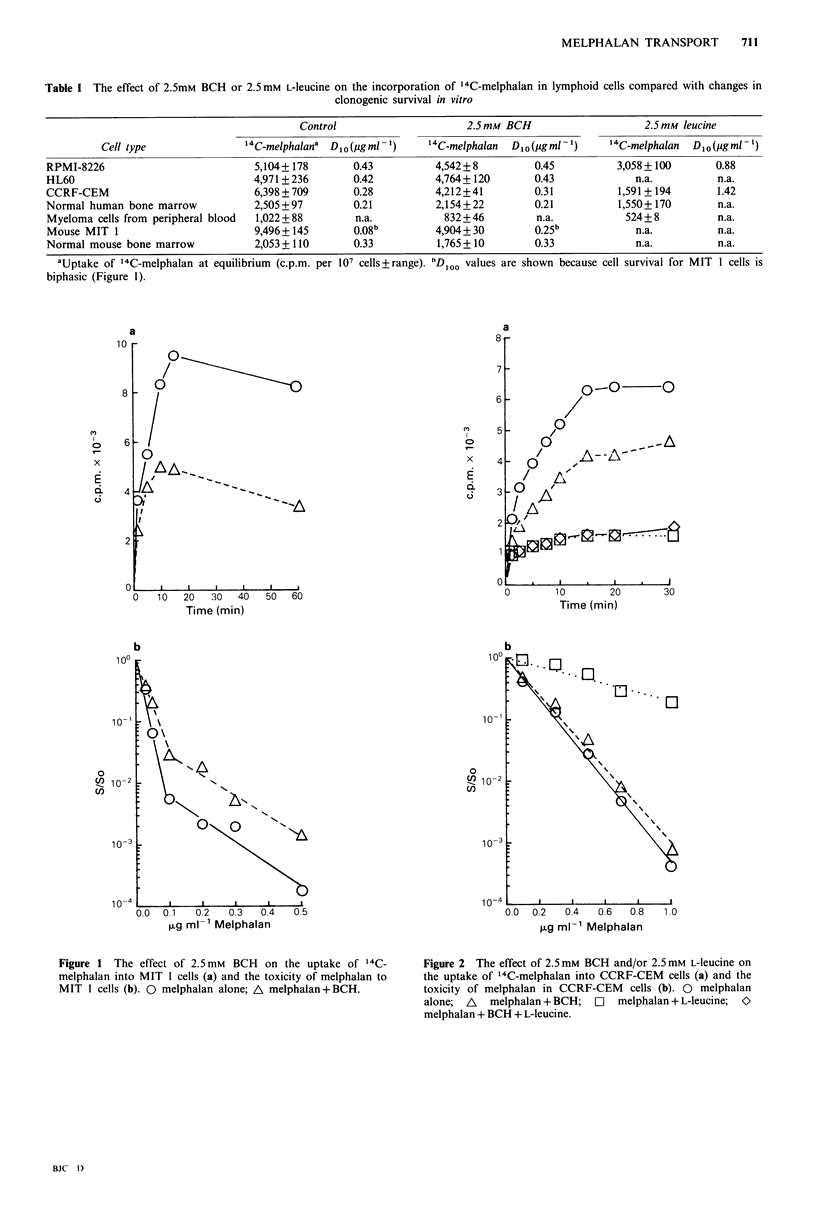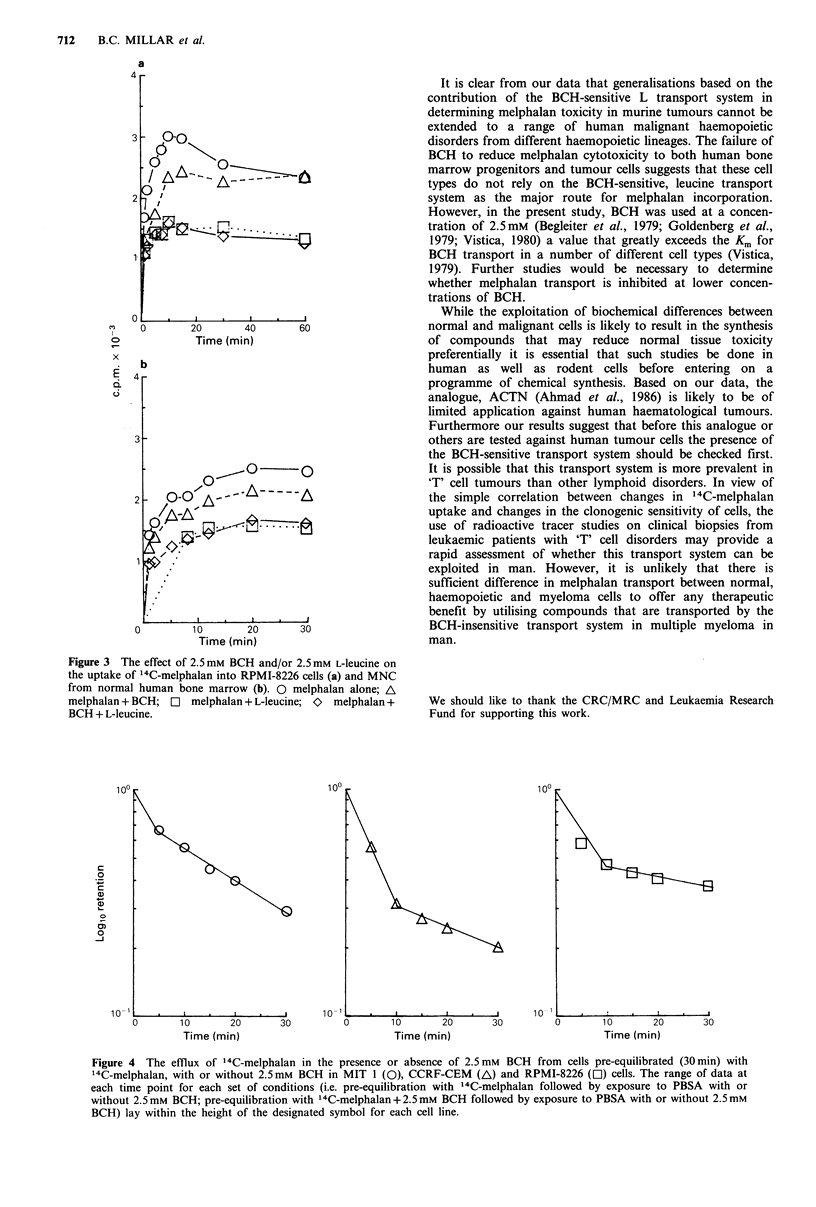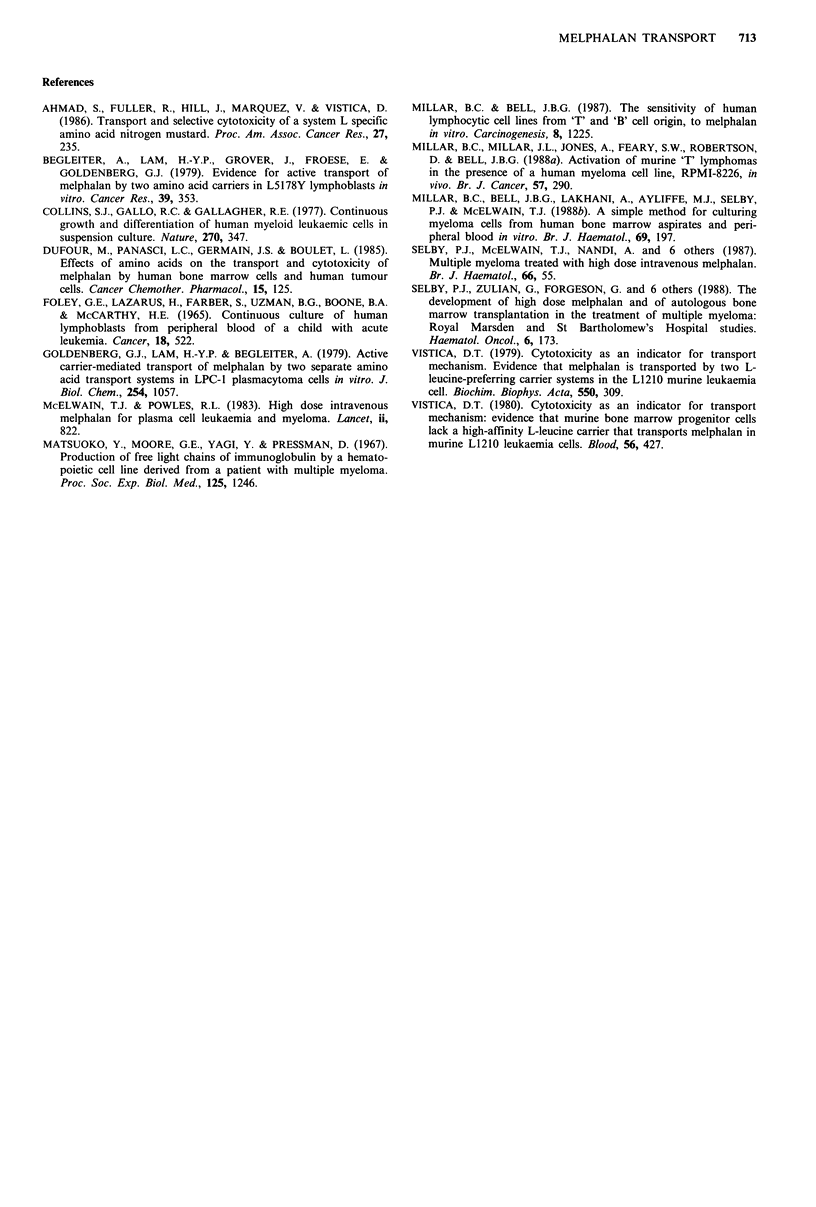# Melphalan transport into human malignant lymphoid cells differs from the murine equivalent in vitro.

**DOI:** 10.1038/bjc.1989.149

**Published:** 1989-05

**Authors:** B. C. Millar, J. A. Maitland, J. L. Millar, J. B. Bell

**Affiliations:** Section of Medicine, Institute of Cancer Research, Sutton, Surrey, UK.


Br. J. Cancer (1989), 59, 710–713 © The Macmillan Press Ltd., 1989

SHORT COMMUNICATION

### Melphalan transport into human malignant lymphoid cells differs from the murine equivalent *in vitro*

B.C. Millar, J.A. Maitland, J.L. Millar & J.B.G. Bell

*Section of Medicine, Institute of Cancer Research, Sutton, Surrey, UK.*

Although melphalan is used extensively as a single agent in the treatment of multiple myeloma, myelosuppression limits the dose of drug used in man particularly in circumstances which cannot include autologous bone marrow transplantation (McElwain & Powles, 1983; Selby *et al.*, 1987, 1988).

Melphalan uptake is mediated by at least two separate amino acid transport systems in murine tumour cells *in vitro* (Begleiter *et al.*, 1979; Goldeburg *et al.*, 1979). One system (L) is inhibited by 2-aminobicyclo-(2,2,1)-heptane-2-carboxylic acid (BCH), a synthetic L-amino acid, and L-leucine. The second transport system is insensitive to BCH and α-aminoisobutyric acid, an analogue of alanine, and also has high affinity for L-leucine (Vistica, 1980). The finding that melphalan is transported into normal murine haemopoietic cells (GM-$CFU_c$) by the BCH-insensitive transport system *in vitro* prompted the synthesis of analogues of nitrogen mustard that are transported by the high affinity BCH-sensitive transport system (Ahmad *et al.*, 1986). Preliminary data in mouse L1210 cells *in vitro* showed that one such compound, the melphalan analogue of 2-amino-2-carboxylic-1,2,3,4-tetrahydronaphthalene (ACTN), improved the therapeutic ratio by selectively increasing drug incorporation into tumour compared to normal murine haemopoietic cells (Ahmad *et al.*, 1986).

Because of the interest in multiple myeloma at the Royal Marsden Hospital, compounds that may reduce myelosuppression by being selectively incorporated into tumour cells may provide additions or alternatives to high dose melphalan in future clinical trials. This study was done to investigate the role of the BCH-sensitive transport system in human lymphoid tumour cell lines compared with normal human bone marrow progenitors (GM-$CFU_c$) with a view to testing compounds such as ACTN for selective activity against myeloma cells taken from clinical biopsies.

Asynchronous cultures of CCRF-CEM (human T) (Foley *et al.*, 1965), HL60 (human myeloid) (Collins *et al.*, 1977), RPMI-8226 (human myeloma) (Matsuoko *et al.*, 1967) and MIT 1 (mouse T cell lymphoma) (Millar *et al.*, 1988*a*) cell lines were propagated by methods described previously (Millar *et al.*, 1988*b*).

Mononuclear cells (MNC) were prepared from bone marrow or peripheral blood as before (Millar *et al.*, 1988*b*). Survival data were determined by exposing cells to different concentrations of melphalan (0.1–1.0 $\mu g\,ml^{-1}$) dissolved in Dulbecco's phosphate-buffered saline (PBSA) in the presence or absence of 2.5 mM BCH or 2.5 mM L-leucine (Dufour *et al.*, 1985) for 1 h at 37°C before plating in semi-solid medium for 2–3 weeks to allow for colony formation (Millar *et al.*, 1988*b*). Dose–response curves were constructed from the mean survival values at each dose point from three separate experiments. $D_{10}$ and $D_{100}$ values were calculated from these pooled data.

The incorporation and efflux of $^{14}$C-melphalan, labelled in the alkylating moiety (0.1 $\mu Ci\,ml^{-1}$; specific activity 10.9 $mCi\,mM^{-1}$; 33.7 $\mu Ci\,mg^{-1}$; Dr R. Engle, NCI, Bethesda, MD, USA) with or without 2.5 mM BCH or 2.5 mM L-leucine was made using methods described previously (Millar & Bell, 1987). Each experiment was done three times for each cell line; examples of single experiments are shown in the text. Efflux was measured in cells which had been equilibrated in the presence or absence of 2.5 mM BCH followed by exposure to PBSA with or without BCH in the surrounding medium. The rate of efflux of $^{14}$C-melphalan was calculated by comparing the c.p.m. in the cell pellets at equilibrium (30 min) with that for each subsequent time point.

The data in Table I confirm the observation that BCH selectively inhibits the incorporation of melphalan into murine tumour cells compared with MNC from normal murine bone marrow (Vistica, 1980). BCH reduced the incorporation of $^{14}$C-melphalan by approximately 50% into MIT 1 cells and this was accompanied by an increase in cell survival (Figure 1). In contrast to previously published results (Dufour *et al.*, 1985) BCH was without effect on the incorporation or toxicity of melphalan to MNC from pooled normal human bone marrow (three samples) (Table I). However, unlike the effect in murine tumour cells, BCH also failed to decrease the toxicity of melphalan to the human malignant lymphoid cell lines, HL60, RPMI-8226 or CCRF-CEM. Both RPMI-8226 and HL60 cells incorporated approximately 90% as much melphalan in the presence of BCH as in its absence. This small decrease was similar to that seen in MNC from normal human bone marrow. Although the incorporation of melphalan into CCRF-CEM cells was decreased by 35% in the presence of BCH, this decrease was not sufficient to detect any changes in clonogenic survival (Figure 2). Furthermore, since the incorporation of melphalan into malignant plasma cells from a patient with plasma cell leukaemia was reduced by only 20% in the presence of BCH, it is unlikely that this decrease in incorporation would be sufficient to permit any changes in cell kill.

In contrast, L-leucine reduced $^{14}$C-melphalan uptake by 40–50% in RPMI-8226 and myeloma cells taken from peripheral blood as well as CCRF-CEM cells, suggesting that in human cells the BCH-insensitive transport system provides the principal route by which melphalan is transported. Although reduction of $^{14}$C-melphalan uptake in cultures of CCRF-CEM cells exposed simultaneously to L-leucine was paralleled by a 5-fold increase in $D_{10}$ for melphalan, toxicity was not abolished. Furthermore, since L-leucine failed to inhibit completely melphalan uptake into either MNC from normal human bone marrow (Figure 3), RPMI-8226 (Figure 3) or CCRF-CEM (Figure 2) cells in the presence of BCH, it seems likely that other transport systems provide a significant contribution towards the uptake of melphalan into human haemopoietic cells.

Examination of the efflux of $^{14}$C-melphalan from MIT 1, CCRF-CEM and RPMI-8226 cells pre-equilibrated with melphalan with or without BCH showed that the differential effect of BCH in decreasing melphalan toxicity towards MIT 1 cells compared with CCRF-CEM and RPMI-8226 cells cannot be accounted for by changes in melphalan efflux (Figure 4).

Correspondence: B.C. Millar, Section of Medicine, F Block, Institute of Cancer Research, Cotswold Road, Sutton, Surrey SM2 5NG.
Received 19 October 1988, and in revised form, 22 December 1988.

**Table I** The effect of 2.5mM BCH or 2.5 mM L-leucine on the incorporation of $^{14}$C-melphalan in lymphoid cells compared with changes in clonogenic survival *in vitro*

| *Cell type* | *Control* | | *2.5 mM BCH* | | *2.5 mM leucine* | |
|---|---|---|---|---|---|---|
| | *$^{14}$C-melphalan*[a] | $D_{10}$ ($\mu g\,ml^{-1}$) | *$^{14}$C-melphalan* | $D_{10}$ ($\mu g\,ml^{-1}$) | *$^{14}$C-melphalan* | $D_{10}$ ($\mu g\,ml^{-1}$) |
| RPMI-8226 | 5,104 ± 178 | 0.43 | 4,542 ± 8 | 0.45 | 3,058 ± 100 | 0.88 |
| HL60 | 4,971 ± 236 | 0.42 | 4,764 ± 120 | 0.43 | n.a. | n.a. |
| CCRF-CEM | 6,398 ± 709 | 0.28 | 4,212 ± 41 | 0.31 | 1,591 ± 194 | 1.42 |
| Normal human bone marrow | 2,505 ± 97 | 0.21 | 2,154 ± 22 | 0.21 | 1,550 ± 170 | n.a. |
| Myeloma cells from peripheral blood | 1,022 ± 88 | n.a. | 832 ± 46 | n.a. | 524 ± 8 | n.a. |
| Mouse MIT 1 | 9,496 ± 145 | 0.08[b] | 4,904 ± 30 | 0.25[b] | n.a. | n.a. |
| Normal mouse bone marrow | 2,053 ± 110 | 0.33 | 1,765 ± 10 | 0.33 | n.a. | n.a. |

[a]Uptake of $^{14}$C-melphalan at equilibrium (c.p.m. per $10^7$ cells ± range). [b]$D_{100}$ values are shown because cell survival for MIT 1 cells is biphasic (Figure 1).

**Figure 1** The effect of 2.5 mM BCH on the uptake of $^{14}$C-melphalan into MIT 1 cells (**a**) and the toxicity of melphalan to MIT 1 cells (**b**). ○ melphalan alone; △ melphalan + BCH.

**Figure 2** The effect of 2.5 mM BCH and/or 2.5 mM L-leucine on the uptake of $^{14}$C-melphalan into CCRF-CEM cells (**a**) and the toxicity of melphalan in CCRF-CEM cells (**b**). ○ melphalan alone; △ melphalan + BCH; □ melphalan + L-leucine; ◇ melphalan + BCH + L-leucine.

Figure 3 The effect of 2.5 mM BCH and/or 2.5 mM L-leucine on the uptake of $^{14}$C-melphalan into RPMI-8226 cells (a) and MNC from normal human bone marrow (b). ○ melphalan alone; △ melphalan + BCH; □ melphalan + L-leucine; ◇ melphalan + BCH + L-leucine.

It is clear from our data that generalisations based on the contribution of the BCH-sensitive L transport system in determining melphalan toxicity in murine tumours cannot be extended to a range of human malignant haemopoietic disorders from different haemopoietic lineages. The failure of BCH to reduce melphalan cytotoxicity to both human bone marrow progenitors and tumour cells suggests that these cell types do not rely on the BCH-sensitive, leucine transport system as the major route for melphalan incorporation. However, in the present study, BCH was used at a concentration of 2.5 mM (Begleiter *et al.*, 1979; Goldenberg *et al.*, 1979; Vistica, 1980) a value that greatly exceeds the $K_m$ for BCH transport in a number of different cell types (Vistica, 1979). Further studies would be necessary to determine whether melphalan transport is inhibited at lower concentrations of BCH.

While the exploitation of biochemical differences between normal and malignant cells is likely to result in the synthesis of compounds that may reduce normal tissue toxicity preferentially it is essential that such studies be done in human as well as rodent cells before entering on a programme of chemical synthesis. Based on our data, the analogue, ACTN (Ahmad *et al.*, 1986) is likely to be of limited application against human haematological tumours. Furthermore our results suggest that before this analogue or others are tested against human tumour cells the presence of the BCH-sensitive transport system should be checked first. It is possible that this transport system is more prevalent in 'T' cell tumours than other lymphoid disorders. In view of the simple correlation between changes in $^{14}$C-melphalan uptake and changes in the clonogenic sensitivity of cells, the use of radioactive tracer studies on clinical biopsies from leukaemic patients with 'T' cell disorders may provide a rapid assessment of whether this transport system can be exploited in man. However, it is unlikely that there is sufficient difference in melphalan transport between normal, haemopoietic and myeloma cells to offer any therapeutic benefit by utilising compounds that are transported by the BCH-insensitive transport system in multiple myeloma in man.

We should like to thank the CRC/MRC and Leukaemia Research Fund for supporting this work.

Figure 4 The efflux of $^{14}$C-melphalan in the presence or absence of 2.5 mM BCH from cells pre-equilibrated (30 min) with $^{14}$C-melphalan, with or without 2.5 mM BCH in MIT 1 (○), CCRF-CEM (△) and RPMI-8226 (□) cells. The range of data at each time point for each set of conditions (i.e. pre-equilibration with $^{14}$C-melphalan followed by exposure to PBSA with or without 2.5 mM BCH; pre-equilibration with $^{14}$C-melphalan + 2.5 mM BCH followed by exposure to PBSA with or without 2.5 mM BCH) lay within the height of the designated symbol for each cell line.

### References

AHMAD, S., FULLER, R., HILL, J., MARQUEZ, V. & VISTICA, D. (1986). Transport and selective cytotoxicity of a system L specific amino acid nitrogen mustard. *Proc. Am. Assoc. Cancer Res.*, **27**, 235.

BEGLEITER, A., LAM, H.-Y.P., GROVER, J., FROESE, E. & GOLDENBERG, G.J. (1979). Evidence for active transport of melphalan by two amino acid carriers in L5178Y lymphoblasts *in vitro*. *Cancer Res.*, **39**, 353.

COLLINS, S.J., GALLO, R.C. & GALLAGHER, R.E. (1977). Continuous growth and differentiation of human myeloid leukaemic cells in suspension culture. *Nature*, **270**, 347.

DUFOUR, M., PANASCI, L.C., GERMAIN, J.S. & BOULET, L. (1985). Effects of amino acids on the transport and cytotoxicity of melphalan by human bone marrow cells and human tumour cells. *Cancer Chemother. Pharmacol.*, **15**, 125.

FOLEY, G.E., LAZARUS, H., FARBER, S., UZMAN, B.G., BOONE, B.A. & McCARTHY, H.E. (1965). Continuous culture of human lymphoblasts from peripheral blood of a child with acute leukemia. *Cancer*, **18**, 522.

GOLDENBERG, G.J., LAM, H.-Y.P. & BEGLEITER, A. (1979). Active carrier-mediated transport of melphalan by two separate amino acid transport systems in LPC-1 plasmacytoma cells *in vitro*. *J. Biol. Chem.*, **254**, 1057.

McELWAIN, T.J. & POWLES, R.L. (1983). High dose intravenous melphalan for plasma cell leukaemia and myeloma. *Lancet*, **ii**, 822.

MATSUOKO, Y., MOORE, G.E., YAGI, Y. & PRESSMAN, D. (1967). Production of free light chains of immunoglobulin by a hematopoietic cell line derived from a patient with multiple myeloma. *Proc. Soc. Exp. Biol. Med.*, **125**, 1246.

MILLAR, B.C. & BELL, J.B.G. (1987). The sensitivity of human lymphocytic cell lines from 'T' and 'B' cell origin, to melphalan *in vitro*. *Carcinogenesis*, **8**, 1225.

MILLAR, B.C., MILLAR, J.L., JONES, A., FEARY, S.W., ROBERTSON, D. & BELL, J.B.G. (1988*a*). Activation of murine 'T' lymphomas in the presence of a human myeloma cell line, RPMI-8226, *in vivo*. *Br. J. Cancer*, **57**, 290.

MILLAR, B.C., BELL, J.B.G., LAKHANI, A., AYLIFFE, M.J., SELBY, P.J. & McELWAIN, T.J. (1988*b*). A simple method for culturing myeloma cells from human bone marrow aspirates and peripheral blood *in vitro*. *Br. J. Haematol.*, **69**, 197.

SELBY, P.J., McELWAIN, T.J., NANDI, A. and 6 others (1987). Multiple myeloma treated with high dose intravenous melphalan. *Br. J. Haematol.*, **66**, 55.

SELBY, P.J., ZULIAN, G., FORGESON, G. and 6 others (1988). The development of high dose melphalan and of autologous bone marrow transplantation in the treatment of multiple myeloma: Royal Marsden and St Bartholomew's Hospital studies. *Haematol. Oncol.*, **6**, 173.

VISTICA, D.T. (1979). Cytotoxicity as an indicator for transport mechanism. Evidence that melphalan is transported by two L-leucine-preferring carrier systems in the L1210 murine leukaemia cell. *Biochim. Biophys. Acta*, **550**, 309.

VISTICA, D.T. (1980). Cytotoxicity as an indicator for transport mechanism: evidence that murine bone marrow progenitor cells lack a high-affinity L-leucine carrier that transports melphalan in murine L1210 leukaemia cells. *Blood*, **56**, 427.